# Quaternary Categorization Strategy for Reconstructing High-Reflectivity Surface in Structured Light Illumination

**DOI:** 10.3390/s23249740

**Published:** 2023-12-10

**Authors:** Bin Xu, Shangcheng Qu, Jinhua Li, Zhiyong Deng, Hongyu Li, Bo Zhang, Geyou Zhang, Kai Liu

**Affiliations:** 1 School of Mechanical Engineering, Sichuan University, Chengdu 610065, China; bin_xu@outlook.com (B.X.); shangcheng_qu@foxmail.com (S.Q.); jinhua07@hotmail.com (J.L.); 2 Nuclear Fuel and Material Institute, Nuclear Power Institute of China, Chengdu 610213, China; dengzhy08@163.com (Z.D.); lihongyuzsdyg@163.com (H.L.); 3 School of Information and Communication Engineering, University of Electronic Science and Technology of China, Chengdu 611731, China; geyouzhang@foxmail.com; 4 College of Electrical Engineering, Sichuan University, Chengdu 610065, China; kailiu@scu.edu.cn

**Keywords:** structured light illumination, high-reflectivity surface, dual projector, 3D measurement

## Abstract

Structured light illumination is widely applied for surface defect detection due to its advantages in terms of speed, precision, and non-contact capabilities. However, the high reflectivity of metal surfaces often results in the loss of point clouds, thus reducing the measurement accuracy. In this paper, we propose a novel quaternary categorization strategy to address the high-reflectivity issue. Firstly, we classify the pixels into four types according to the phase map characteristics. Secondly, we apply tailored optimization and reconstruction strategies to each type of pixel. Finally, we fuse point clouds from multi-type pixels to accomplish precise measurements of high-reflectivity surfaces. Experimental results show that our strategy effectively reduces the high-reflectivity error when measuring metal surfaces and exhibits stronger robustness against noise compared to the conventional method.

## 1. Introduction

Three-dimensional measurement technology is widely applied in significant domains [1], such as aviation, aerospace, and nuclear energy, primarily facilitating tasks such as defect detection and dimensional measurement [2,3,4,5]. This technology encompasses stereovision [6], line laser scanning [7], and structured light illumination (SLI). Among these methods, SLI stands out for its higher applicability and growth potential due to its advantages in terms of high speed, high precision, and cost-effectiveness. However, within industrial measurement, conventional SLI techniques are primarily suited to measuring diffuse reflective surfaces [8]. When it comes to metal surfaces, there is a risk of image oversaturation, primarily in regions with high reflectivity that exceeds the camera’s intensity response range. The image oversaturation can pose challenges in obtaining effective 3D reconstruction results [9]. One approach to addressing this problem is to apply a diffuse reflective layer onto high-reflectivity surfaces. However, it is crucial to note that the thickness of the layer can decrease the measurement accuracy [10], and the layer may cause corrosion to the measured surface. Consequently, this approach is unsuitable for precision 3D measurement scenarios. Furthermore, it is essential to note that in complex industrial scenarios, there is an increase in noise within camera images. Preprocessing [11,12] of these images can be performed to mitigate errors in point cloud reconstruction.

To address the 3D measurement challenges posed by high-reflectivity surfaces, researchers have explored various approaches to enhance conventional SLI techniques [13]. One approach is to adjust the projection strategy, which can be categorized into two types. The first type involves capturing multiple sets of fringe images with different exposure times and subsequently merging them into a single set. This helps to overcome issues related to the saturation and contrast problems resulting from varying reflectivity [14,15]. The second type entails obtaining surface reflectivity information through pre-projection and subsequently adjusting the overall projection intensity according to this information [16], or dynamically modifying the projection intensity [17] based on various reflectivity areas. These strategies effectively mitigate the occurrence of saturated pixels and prove to be beneficial in measuring high-reflectivity surfaces. However, they require the capture of multiple images and adjustments to the camera exposure settings, which may result in complex scanning processes and limited robustness. The second approach is to utilize polarization characteristics, as specular reflection exhibits stronger polarization in comparison to diffuse reflection. By utilizing polarizers on both the camera and projector [18,19], it becomes possible to reduce the impact of specular reflection in saturated areas [20]. Nonetheless, this approach also decreases the intensity in non-saturated areas, potentially reducing the measurement accuracy. The third approach employs the properties of specular reflection, where the location of saturated regions is closely linked to the incident angle of projected light. Therefore, employing multiple camera viewpoints [8,21] or projection directions [22] ensures that at least one of them captures valid images for the measurement of the saturated areas in other viewpoints. This approach does not decrease the accuracy in measuring non-saturated areas and results in high-quality overall measurements. In the multi-camera system mentioned above, the camera can be interchanged with the projector to form a multi-projector system [23,24]. This configuration not only enables measurements of high-reflectivity surfaces but also mitigates shadow issues [25,26,27].

**Figure 1:**
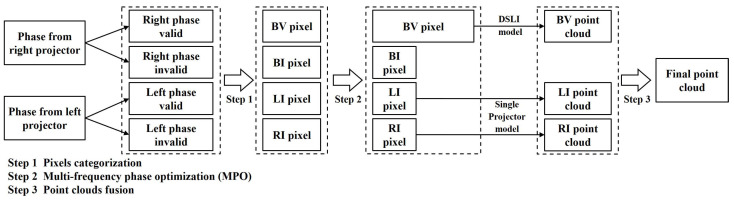
Flow chart of quaternary categorization strategy.

In this paper, we propose a quaternary categorization measurement strategy for high-reflectivity surfaces, which relies on two projectors and one camera. To enhance the measurement accuracy, we align the projection fringe direction perpendicular to the system baseline [28]. The core concept of the proposed method is to obtain two sets of phase information through two projectors. According to the left phase map and right phase map, camera pixels are classified into four categories by phase validity detection: both valid (BV), both invalid (BI), right invalid (RI), and left invalid (LI). For BI, RI, and LI pixels, with the aim of enhancing the measurement accuracy, we introduce a multi-frequency phase optimization (MPO) method to optimize a portion of these pixels into BV pixels. Regarding BV pixels, we propose the dual-projector structured light illumination (DSLI) model to compute 3D coordinates. The DSLI model surpasses conventional the single-projector model in terms of reconstruction accuracy, as shown in Equation (7) in Ref. [29]. For RI and LI pixels, we employ the single-projector model to compute the 3D coordinates. The very few BI pixels are directly eliminated. Ultimately, these three categories of point clouds are combined to generate the final point cloud of the high-reflectivity surface. The specific flow of the strategy is illustrated in Figure 1.

The remainder of the paper is organized as follows. The DSLI model is introduced in Section 2.1, the pixel categorization strategy is described in Section 2.2, the MPO method is presented in Section 2.3, and the point cloud fusion strategy is shown in Section 2.4. Experimental designs and results are discussed in Section 3. Conclusions and future work are summarized in Section 4.

## 2. Methods

### 2.1. DSLI Model for 3D Reconstruction

Given that we project fringe patterns onto a highly reflective surface, the specular reflection ray reaching the camera can induce an oversaturation phenomenon in the image. Hence, this oversaturation leads to the loss of encoded information and the presence of outliers in the point cloud. We propose employing two projectors to conduct 3D scanning successively from different directions. Due to the small scattering angle of specular reflection, the modulated patterns obtained from different projectors may not saturate at the same measured point.

**Figure 2:**
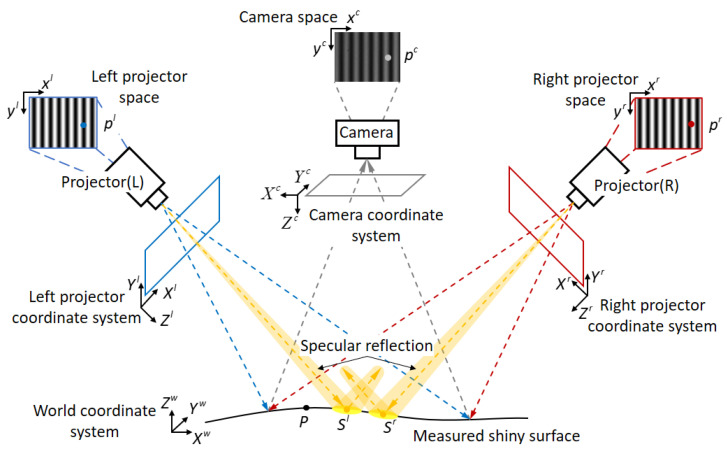
DSLI model and explanation of each symbol.

In the DSLI model, depicted in Figure 2, $S^{l}$ and $S^{r}$ represent saturated areas resulting from two different projections. A pinhole camera is employed, where $\left(X^{w},Y^{w},Z^{w}\right)$ denotes the coordinate of point *P* in the world coordinate system, $\left(X^{c},Y^{c},Z^{c}\right)$ represents the coordinate of the same point *P* in the camera coordinate system, and $p^{c}\left(x^{c},y^{c}\right)$ signifies the corresponding coordinate of $\left(X^{c},Y^{c},Z^{c}\right)$ within the camera space. $p^{c}\left(x^{c},y^{c}\right)$ and $\left(X^{w},Y^{w},Z^{w}\right)$ can be related as follows:

(1) $$s^{c}{\left[\begin{array}{ccc} x^{c} & y^{c} & 1 \end{array}\right]}^{⊤}=M^{c}{\left[\begin{array}{cccc} X^{w} & Y^{w} & Z^{w} & 1 \end{array}\right]}^{⊤},$$

where $s^{c}$ is an arbitrary non-zero scalar, and $M^{c}$ is the 3 × 4 calibration matrix of the camera. Equation (1) represents the perspective transformation of 3D coordinate $\left(X^{w},Y^{w},Z^{w}\right)$ into 2D coordinate $\left(x^{c},y^{c}\right)$. The two projectors can be regarded as the inverse of the camera. Equation (1) can be extended as [23]

(2) $$\left\{\begin{array}{c} s^{c}{\left[\begin{array}{ccc} x^{c} & y^{c} & 1 \end{array}\right]}^{⊤}=M^{c}{\left[\begin{array}{cccc} X^{w} & Y^{w} & Z^{w} & 1 \end{array}\right]}^{⊤}\\ s^{r}{\left[\begin{array}{ccc} x^{r} & y^{r} & 1 \end{array}\right]}^{⊤}=M^{r}{\left[\begin{array}{cccc} X^{w} & Y^{w} & Z^{w} & 1 \end{array}\right]}^{⊤}\\ s^{l}{\left[\begin{array}{ccc} x^{l} & y^{l} & 1 \end{array}\right]}^{⊤}=M^{l}{\left[\begin{array}{cccc} X^{w} & Y^{w} & Z^{w} & 1 \end{array}\right]}^{⊤} \end{array}\right.,$$

where $s^{r}$, $x^{r}$, $y^{r}$, $M^{r}$, $s^{l}$, $x^{l}$, $y^{l}$, and $M^{l}$ are the parameters of the right and left projectors; $M^{c}$, $M^{r}$, $M^{l}$ are obtained by system calibration. During the calibration process, we divide the DSLI model into two single-projector models and calibrate each of them separately [30]. We reorganize Equation (2) and have

(3) $$A{\left[\begin{array}{ccc} X^{w} & Y^{w} & Z^{w} \end{array}\right]}^{⊤}=B,$$

with

(4) $$A=\left[\begin{array}{ccc} m_{11}^{c}-x^{c}m_{31}^{c} & m_{12}^{c}-x^{c}m_{32}^{c} & m_{13}^{c}-x^{c}m_{33}^{c}\\ m_{21}^{c}-y^{c}m_{31}^{c} & m_{22}^{c}-y^{c}m_{32}^{c} & m_{23}^{c}-y^{c}m_{33}^{c}\\ m_{21}^{r}-y^{r}m_{31}^{r} & m_{22}^{r}-y^{r}m_{32}^{r} & m_{23}^{r}-y^{r}m_{33}^{r}\\ m_{21}^{l}-y^{l}m_{31}^{l} & m_{22}^{l}-y^{l}m_{32}^{l} & m_{23}^{l}-y^{l}m_{33}^{l} \end{array}\right],$$

and

(5) $$B={\left[\begin{array}{cccc} x^{c}m_{34}^{c}-m_{14}^{c} & y^{c}m_{34}^{c}-m_{14}^{c} & y^{r}m_{34}^{r}-m_{14}^{r} & y^{l}m_{34}^{l}-m_{14}^{l} \end{array}\right]}^{⊤},$$

where $m_{ij}^{c}\in M^{c}$, $m_{ij}^{r}\in M^{r}$ and $m_{ij}^{l}\in M^{l}$, $i\in \left[1,3\right]$. Finally, our dual-projector model computes 3D coordinates as

(6) $${\left[\begin{array}{ccc} X^{w} & Y^{w} & Z^{w} \end{array}\right]}^{⊤}={\left(A^{⊤}A\right)}^{-1}\left(A^{⊤}B\right).$$

We conduct 3D scanning with sinusoidal phase-shifting fringe patterns. After the images with deforming patterns are captured by the camera, the images can be expressed as

(7) Inr=Ar+Brcos(ϕr−2πn2πnNN)Inl=Al+Blcos(ϕl−2πn2πnNN),

where, for each camera coordinate $\left(x^{c},y^{c}\right)$, $I_{n}^{r}$ and $I_{n}^{l}$ are the intensities from the right and left projectors, $A^{r}$ and $A^{l}$ are the background values, $B^{r}$ and $B^{l}$ are the modulation values, $\phi^{r}$ and $\phi^{l}$ are the phases, *n* is the phase shift, and *N* is the total number of phase shifts. The parameters $y^{r}$ and $y^{l}$, needed in Equation (6) for point cloud computing, can be obtained by computing phases with

(8) ϕr=tan−1∑n=0N−1Inrsin(2πn2πnNN)∑n=0N−1Inrcos(2πn2πnNN)ϕl=tan−1∑n=0N−1Inlsin(2πn2πnNN)∑n=0N−1Inlcos(2πn2πnNN),

where $\phi^{r}$ and $\phi^{l}$ are the wrapped phase that can be unwrapped as $\Phi^{r}$ and $\Phi^{l}$. $y^{r}$ and $y^{l}$ can be expressed as [30]

(9) yr=ΦrHpΦrHp2π2πyl=ΦlHpΦlHp2π2π,

where $H^{p}$ is the vertical resolution of the projectors. By introducing two sets of phase information, our DSLI model constructs a novel overdetermined system of equations with four expressions and three unknowns. In contrast to the single-projector model, our method incorporates an additional constraint from the second projector, enhancing the robustness and accuracy of the computed 3D coordinates [31]. This improvement will be demonstrated in the experiments.

### 2.2. Pixel Categorization Based on Phase Validity Detection

**Figure 3:**
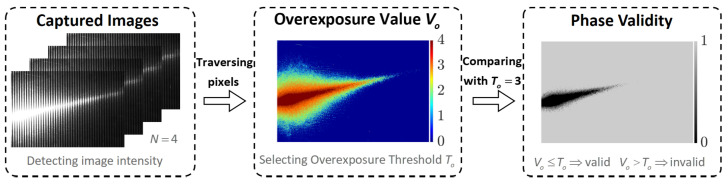
Detecting the phase validity from captured images.

For high-reflectivity surfaces, camera image oversaturation leads to invalid phase values, which can be detected as illustrated in Figure 3. According to Equation (8), the oversaturation of the camera image intensity $I_{n}$ results in the inaccurate detection of the phase ϕ. Consequently, we detect the phase validity by processing the image intensity. In the case of a set of N-step phase-shift images, we traverse each pixel, counting the occurrences of saturated images for individual pixels, denoted as the overexposure value $V_{o}$, which can be expressed as

(10) $$V_{o}\left(x^{c},y^{c}\right)=\frac{1}{2}\sum_{n=1}^{N}\left[\mathrm{sgn}\left(I_{n}^{c}\left(x^{c},y^{c}\right)-255\right)+1\right],$$

where $\mathrm{sgn}\left(x\right)=\left\{\begin{array}{c} -1,\\ 0,\\ 1, \end{array}\right.\begin{array}{c} \left(x<0\right)\\ \left(x=0\right)\\ \left(x>0\right) \end{array}$. Subsequently, we establish an overexposure threshold $T_{o}$ (0 < $T_{o}$ < *N*) and employ a comparison between the overexposure value and the overexposure threshold to assess the validity of the phase for each pixel. Typically, we set $T_{o}$ as N−1 based on experimental experience. The detection result *K* can be expressed as

(11) $$K\left(x^{c},y^{c}\right)=\left\{\begin{array}{c} \begin{array}{cc} 1, & V_{o}\left(x^{c},y^{c}\right)\leq T_{o} \end{array}\\ \begin{array}{cc} 0, & V_{o}\left(x^{c},y^{c}\right)>T_{o} \end{array} \end{array}\right.,$$

Taking the four-step phase shift shown in Figure 3 as an example, the overexposure value for each pixel ranges from 0 to 4. By setting the overexposure threshold to 3, we can then derive the phase validity detection for each pixel.

For each pixel, there are two phases originating from the right projector and the left projector. Employing Equation (11), we derive detection results $K^{r}$ and $K^{l}$, respectively. These results are then summed to obtain the detection result $K^{d}$ for the dual-projector system. Based on $K^{d}$, $K^{r}$, and $K^{l}$, we classify all pixels into four distinct categories.

(1) Pixels with $K^{d}=2$ are classified as BV pixels, and their 3D coordinates are calculated using the DSLI model.
(2) Pixels with $K^{d}=1$ and $K^{r}=1$ are classified as LI pixels, and their 3D coordinates are calculated using the single-projector model.
(3) Pixels with $K^{d}=1$ and $K^{l}=1$ are classified as RI pixels, and their 3D coordinates are calculated using the single-projector model.
(4) Pixels with $K^{d}=0$ are classified as BI pixels, and it is not possible to calculate their 3D coordinates.

### 2.3. MPO Based on Phase Validity Detection

**Figure 4:**
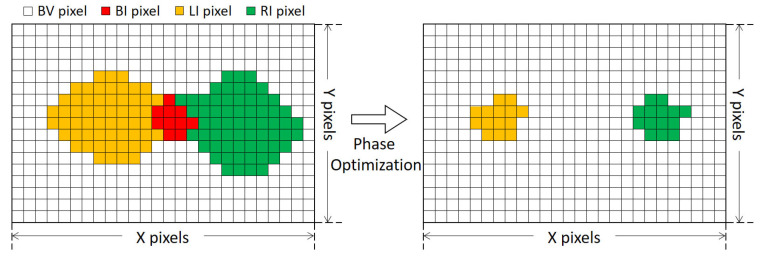
The change in pixel categorization before and after phase optimization.

Due to variations in the reconstruction models, BV pixels exhibit superior point cloud quality compared to LI and RI pixels. Conversely, BI pixels lack valid phase information, leading to gaps in the reconstructed point cloud data. To enhance the reconstruction quality, it is essential to optimize these invalid phases, consequently increasing the count of BV pixels while decreasing the occurrence of BI, LI, and RI pixels, as illustrated in Figure 4.

In multi-frequency phase-shifted structured light, multiple sets of projection patterns with distinct frequencies are employed. The number of BV pixels varies among these different sets of patterns. The variation in the number of BV pixels can mainly be attributed to the following two factors. (1) There are substantial frequency disparities between high-frequency and fundamental-frequency patterns. As the frequency of the fringe pattern decreases, the size of its high-intensity regions also grows, leading to larger areas of saturation, and a decrease in BV pixels. (2) To enhance the phase accuracy, high-frequency patterns typically employ a larger number of phase-shift steps [32]. A larger number of phase-shift steps reduces the impact of saturated images on the phase, leading to an increase in BV pixels.

**Figure 5:**
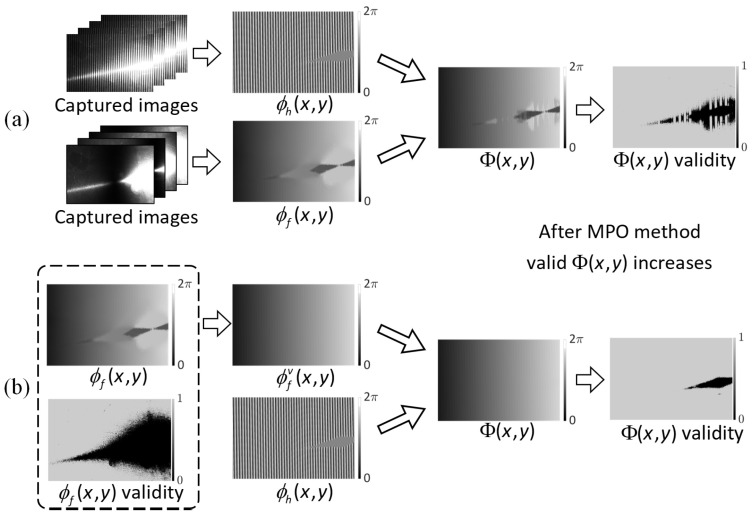
The process of phase unwrapping: (**a**) without MPO method, (**b**) with MPO method.

In multi-frequency phase unwrapping, the fundamental-frequency phase is utilized to compute the high-frequency wrapped phase, resulting in the absolute phase, denoted as Φ. This process can be represented as

(12) $$\Phi =\frac{\phi_{h}+2\pi \mathrm{round}\left(\frac{f\phi_{f}-\phi_{h}}{2\pi }\right)}{f},$$

where $\phi_{h}$ and $\phi_{f}$ are the high-frequency and fundamental-frequency wrapped phases, *f* is the frequency of the high-frequency phase, and round(·) is the symbol of rounding. In the process shown in Equation (12), only when both the high-frequency phase and the fundamental-frequency phase are in the BV state, the absolute phase can display the BV state. Therefore, we propose the MPO method, as illustrated in Figure 5, to optimize the fundamental-frequency phase, ensuring that the BV count in the absolute phase is maximized.

The fundamental-frequency images are processed according to the phase validity detection method demonstrated in Equation (11), resulting in a binary image labeled as *D*. In this binary image, pixels with valid phases are denoted as 1, while pixels with invalid phases are denoted as 0. To identify the boundaries of phase-invalid connected regions, we apply image erosion to the image *D*, which is defined as

(13) $$\left\{\begin{array}{c} D⊖E=\left\{F|{\left(E\right)}_{F}\subseteq D\right\}\\ P=D-F \end{array}\right.,$$

where *E* represents the erosion kernel, *F* represents the binary image after erosion, and *P* represents the difference image between the original image and the eroded image. Pixels with $P\left(x,y\right)=1$ represent the outer boundaries of the phase-invalid connected regions. The phases of the outer boundary pixels are employed for surface fitting, and the fitted surface serves as an interpolation for the phase-invalid regions. This process allows us to derive a valid fundamental-frequency phase denoted as

(14) $$\phi_{f}^{v}\left(x,y\right)=\left\{\begin{array}{c} \phi_{f}\left(x,y\right),\\ ax^{2}+by^{2}+cx+dy+e, \end{array}\begin{array}{cc} \; & \left(D\left(x,y\right)=1\right)\\ \; & \left(D\left(x,y\right)=0\right) \end{array}\right.,$$

where *a* through *e* denote fitting parameters, typically estimated using the least squares method [33,34]. The estimation involves minimizing the following loss function *L* to obtain the best-fitting parameters:

(15) $$L\left(a,b,c,d,e\right)=\sum_{p=1}^{P_{n}}{\left(\phi_{f}\left(x^{p},y^{p}\right)-\left({\left(ax^{p}\right)}^{2}+{\left(by^{p}\right)}^{2}+cx^{p}+dy^{p}+e\right)\right)}^{2},$$

where $P_{n}$ represents the total number of $P\left(x,y\right)=1$, and $\left(x^{p},y^{p}\right)$ denotes the image coordinates of the pth pixel. Next, we substitute the original fundamental-frequency phase in Equation (12) with $\phi_{f}^{v}\left(x,y\right)$, enabling us to obtain

(16) $$\Phi \left(x,y\right)=\frac{\phi_{h}\left(x,y\right)+2\pi \mathrm{round}\left(\frac{f\phi_{f}^{v}\left(x,y\right)-\phi_{h}\left(x,y\right)}{2\pi }\right)}{f},$$

where, if there are only two distinct sets of phase-shifting frequencies, Φ(x,y) represents the ultimate absolute phase. However, when three or more sets of phase-shifting frequencies are involved, Φ(x,y) serves as the new fundamental-frequency phase. This iterative process is repeated until the absolute phase of the highest frequency is successfully calculated.

### 2.4. Point Cloud Fusion Based on Pixel Categorization

**Figure 6:**
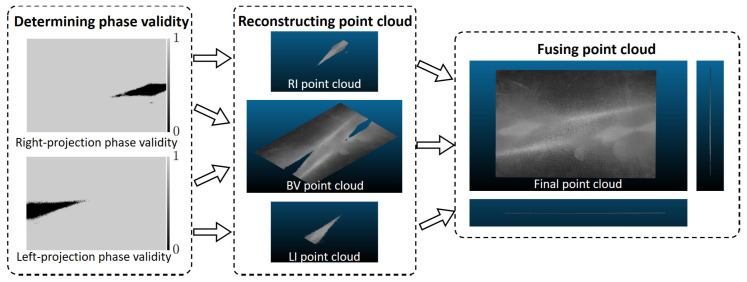
The process of obtaining the final point cloud.

**Figure 7:**
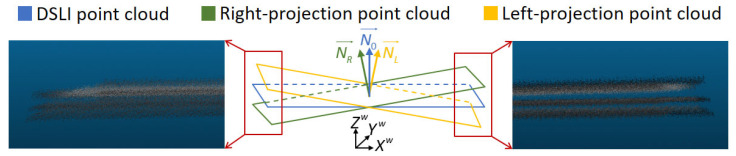
Deviation between different point clouds.

**Figure 8:**
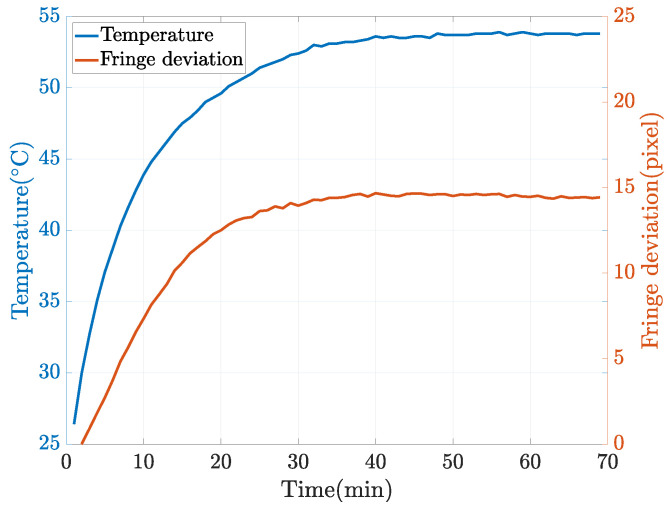
The curve graph of fringe deviation and temperature over time (the projector model is the LightCrafter 4500).

Following the pixel categorization method outlined in Section 2.2, we acquire three distinct sets of point cloud data for the high-reflectivity surface under measurement. These sets are denoted as the BV point cloud, RI point cloud, and LI point cloud, respectively. As depicted in Figure 6, we employ the RI and LI point clouds to address gaps in the BV point cloud. Ideally, with the same calibration information, these three sets of point clouds should align seamlessly and be directly fused. However, due to the thermal noise and lens distortion present in the projector, deviations occur in the reconstructed point clouds across different projection states, as illustrated in Figure 7. Thermal noise [35], primarily attributed to temperature fluctuations within the DLP projection system, directly causes an overall shift in fringe patterns. The relationship between temperature and fringe deviation is illustrated in Figure 8. Hence, we stabilize the operating temperature of the projectors to avoid the influence of thermal noise. Lens distortion can cause deformation in the reconstructed point clouds of planes, such as warping [36]. The varying distortion among different lenses results in varying deformation in the point clouds.

**Figure 9:**
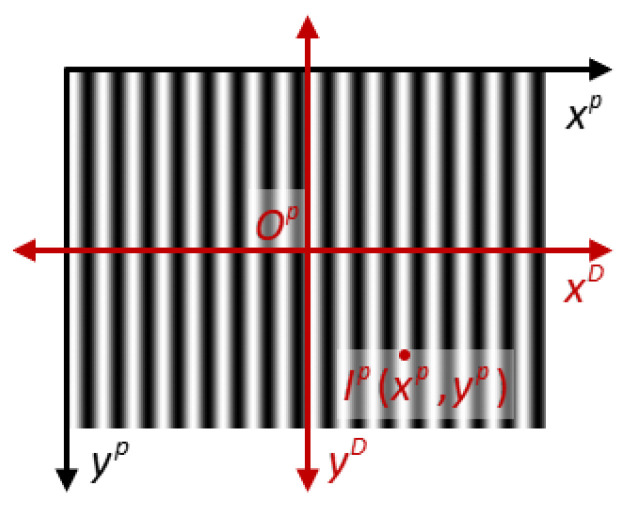
Decomposition of the projected fringe pattern distortion.

The projector lens introduces distortion to the projected fringe pattern, and by investigating the impact of this distortion on the fringe pattern, we can propose suitable solutions. In Figure 9, $I^{p}\left(x^{p},y^{p}\right)$ represents the intensity of the projected fringe pattern, and the distortion is centered on the optical origin of the projector, denoted as $O^{p}$. This distortion is decomposed into two directions, which are $x^{D}$ and $y^{D}$. The pixel axes of the projection pattern, $x^{p}$ and $y^{p}$, are parallel to $x^{D}$ and $y^{D}$, respectively. The fringe pattern exhibits sinusoidal variation along the $x^{p}$ direction, with no variation along the $y^{p}$ direction. Due to the distortion in the $x^{D}$ direction, the intensity of the actual pattern, $I_{a}^{p}$, differs from the intensity of the ideal pattern, $I_{i}^{p}$. Consequently, this distortion leads to deviations in the solved phase, resulting in changes in the correspondence between the phase and 3D coordinates. Conversely, in the $y^{D}$ direction, there is a minimal difference between $I_{a}^{p}$ and $I_{i}^{p}$, thus having no effect on the 3D coordinates. Finally, as depicted in Figure 7, due to variations in the distortion parameters of the two projectors, the $Z^{w}$ coordinates exhibit relative tilting and sinking along the $X^{w}$ direction. To simplify the calculation, we approximate the deviation between the point clouds as a rigid deformation, which is subsequently corrected using a calibration matrix. This correction process is outlined as

(17) $${\left[\begin{array}{ccc} X_{c}^{w} & Y_{c}^{w} & Z_{c}^{w} \end{array}\right]}^{⊤}=R_{c}{\left[\begin{array}{ccc} X^{w} & Y^{w} & Z^{w} \end{array}\right]}^{⊤}+T_{c},$$

where $\left(X^{w},Y^{w},Z^{w}\right)$ represents the original 3D coordinate, $\left(X_{c}^{w},Y_{c}^{w},Z_{c}^{w}\right)$ represents the corrected 3D coordinates, and the calibration matrix is composed of a rotation matrix, $R_{c}$, and a translation matrix, $T_{c}$. For the rotation matrix $R_{c}$, we employ a checkerboard as a calibration target. We reconstruct three distinct point clouds and derive their normal vectors, named $\vec{N_{0}}$, $\vec{N_{R}}$, and $\vec{N_{L}}$ (as depicted in Figure 7). Utilizing the Rodrigues rotation formula, we calculate the matrix $R_{c}$ based on $\vec{N_{0}}$ and another normal vector. Following the rotation, we derive the corner coordinates of the calibration target and subsequently calculate the average deviation between these corner coordinates in the DSLI point cloud and another point cloud. This average deviation provides the specific values for the translation matrix $T_{c}$.

Following the correction of the point clouds, we minimize the discrepancies between them, enabling the fusion of the different point clouds. As illustrated in Figure 6, we fuse the three sets of point clouds to obtain the final point cloud.

## 3. Experiments

**Figure 10:**
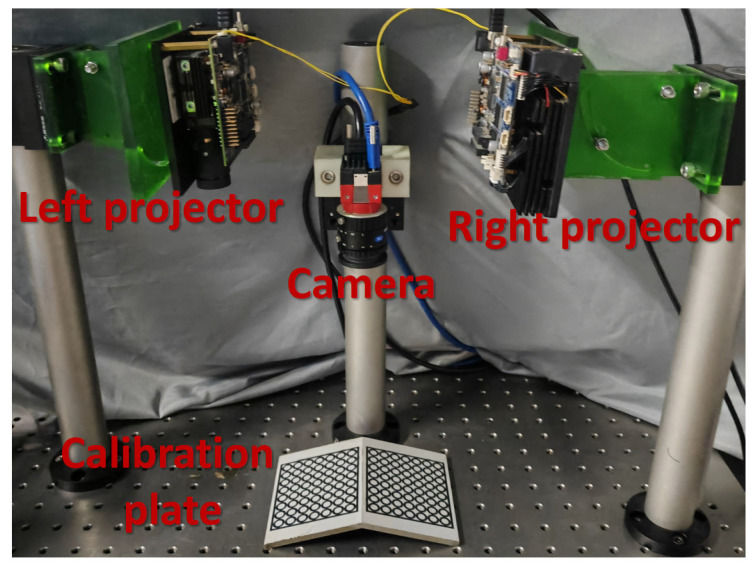
Experimental system setup.

Our experimental system consists of two Texas Instruments (Dallas, TX, USA) LightCrafter 4500 DLP projectors with a resolution of 912 × 1140 and an Allied Vision (Stadtroda, Germany) Alvium 1800U-2050m camera with a resolution of 5496 × 3672, as shown in Figure 10. We align the fringe direction perpendicular to the system baseline to ensure system accuracy [28]. In our subsequent experiments, we utilize three distinct frequencies to obtain the wrapped phase, subsequently applying the conventional temporal phase unwrapping method to obtain the unwrapped phase. Furthermore, we specify the parameters for the fringe patterns as follows: the spatial frequency of patterns is denoted as $f=\{f_{l},f_{m},f_{h}\}$, where $f_{l}$ represents the fundamental frequency, $f_{m}$ represents the intermediate frequency, and $f_{h}$ represents the high frequency; the phase shift number of the patterns is denoted as $N=\{N_{l},N_{m},N_{h}\}$, where $N_{l}$, $N_{m}$, and $N_{h}$ correspond to the total number of phase shifts for each frequency, respectively.

### 3.1. Measuring Standard Plane

To evaluate the proposed DSLI model, we conducted a comparative analysis with the single-projection model. Both methods were employed to scan a standardized plane measuring 50 mm × 70 mm, with a flatness specification better than 1 μm. In this experimental setup, we set all frequency parameters as f={1,8,64}. However, for the phase shift parameters, we employed two different parameters, which were $N_{1}=\left\{4,4,12\right\}$ and $N_{2}=\left\{4,4,120\right\}$. In the process of solving the phase, it is well established that increasing the number of phase shifts can effectively suppress noise [32]. Hence, we consider the 3D results obtained with $N_{2}=\left\{4,4,120\right\}$ as the reference values, while those acquired with $N_{1}=\left\{4,4,12\right\}$ are regarded as the measured values. Due to the projection limit of 128 patterns imposed by the LightCrafter 4500, the option of $N_{2}$ could only be set as {4,4,120}.

**Figure 11:**
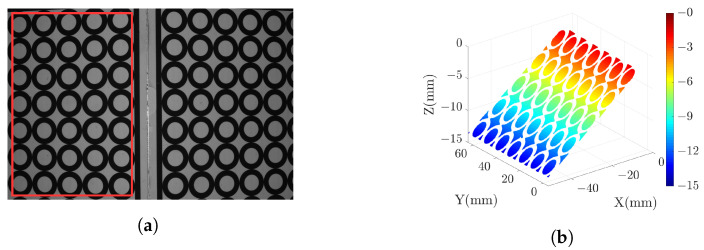
Measured result of the standard plane with $N_{2}=\left\{4,4,120\right\}$: (**a**) grayscale image and the selected measured area, (**b**) point cloud map of the selected area (the points with high gray values are eliminated).

**Figure 12:**
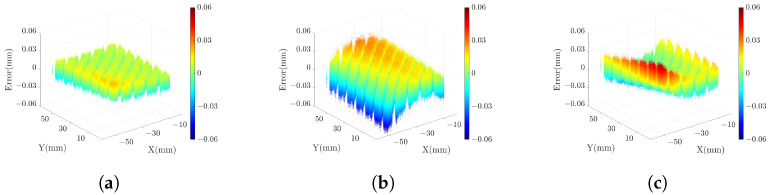
The scatter plots of errors for each point in the standard plane from (**a**) DSLI model, (**b**) SRP model, and (**c**) SLP model.

**Figure 13:**
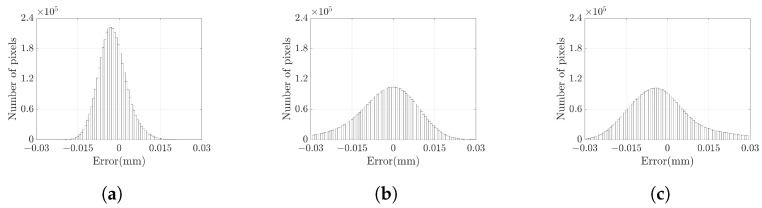
The histograms of error distribution in the standard plane from (**a**) DSLI model, (**b**) SRP model, and (**c**) SLP model.

The grayscale image of the standard plane is displayed in Figure 11 (**a**), with the pixels inside the red box identified as the measured area. High-gray-value pixels within the grayscale image are excluded from consideration. The selected 3D point cloud is visualized in Figure 11 (**b**). For measuring the standard plane, we use the DSLI model, single-right-projector (SRP) model, and single-left-projector (SLP) model using two different sets of parameters. This results in three sets of reference values and three sets of measured values. To quantify the measurement accuracy, we define the error as the Z-axis distance between the measured value and the reference value and calculate the error for each point based on the three different models. Table 1 lists the mean absolute error (MAE), root mean squared error (RMSE), and peak-to-valley (PV) of the point cloud for various models. The results demonstrate that the RMSE can be reduced by more than 50% in the DSLI model. Figure 12 illustrates the errors for individual points across different models, while Figure 13 depicts the error distributions under varying model conditions. These figures reveal that the errors in the DSLI model are primarily confined to the range of 0–0.015 mm, whereas certain errors in the SRP and SLP models significantly exceed 0.015 mm.

According to the experimental results when measuring the standard plane, it can be concluded that the DSLI model exhibits superior phase sensitivity, leading to an effective enhancement in the accuracy of the 3D coordinates. Therefore, the MPO method in Section 2.3 can effectively improve the point cloud accuracy.

### 3.2. Measuring Precision Microgrooves

**Figure 14:**
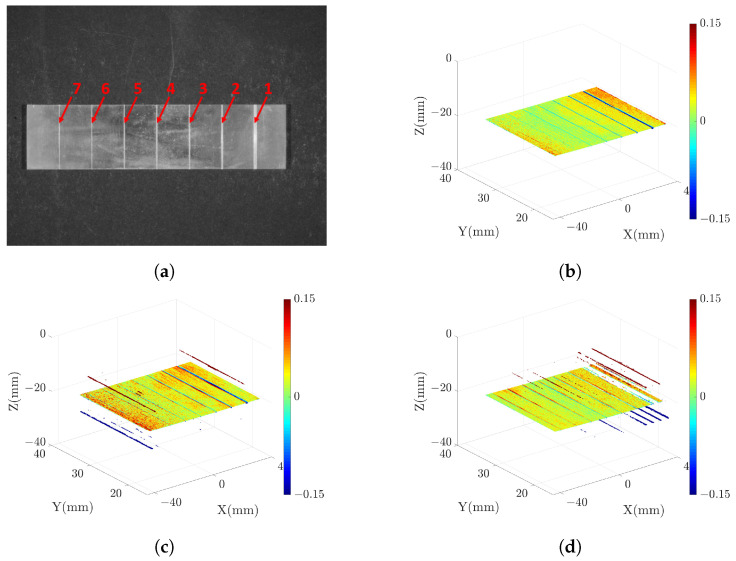
Measured results of microgrooves: (**a**) grayscale image, (**b**) point cloud in proposed strategy, (**c**) point cloud in SRP model, (**d**) point cloud in SLP model. In (**b**–**d**), the color represents the distance from the point to the datum surface.

In order to verify the measurement capability of the proposed strategy, we measure a high-reflectivity copper block with seven precision microgrooves. These microgrooves are machined with accuracy exceeding 1 μm, and the surface of the block is prone to specular reflection. Arranged from right to left on the copper block are seven microgrooves of varying sizes. we employ patterns with f={1,8,64}, N={4,4,12} to measure these microgrooves. The results of these measurements are illustrated in Figure 14. In Figure 14 (**b**)–(**d**), we fit all the points to a datum surface and use distinct colors to represent the distances between the points and the datum surface. It is evident that, in comparison to the single-projector model, the proposed strategy effectively mitigates the issue of excessive outliers arising from specular reflection.

**Figure 15:**
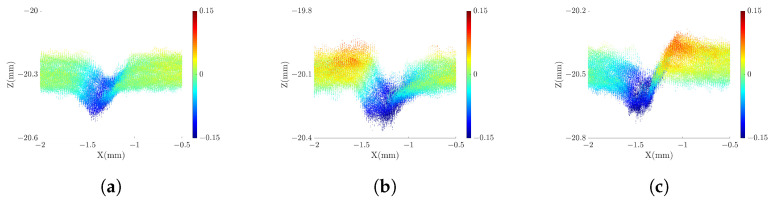
Measured results of microgroove number 4: (**a**) point cloud in proposed strategy, (**b**) point cloud in SRP model, and (**c**) point cloud in SLP model. The color represents the distance from the point to the datum surface.

When calculating the depth of the microgroove, we perform plane fitting for the points on both sides of the microgroove. The average distance between the microgroove points and the fitting plane is then determined as the depth measured value (DMV). The horizontal accuracy of the point cloud is influenced by both the z-direction accuracy and the camera parameters [29]. The z-direction accuracy can be directly assessed through the DMV. We define the difference between the DMV and the depth ground truth as the measurement error, which serves as an indicator of the system’s measurement capabilities. In Figure 15, we present the side-view point cloud of microgroove number 4, clearly demonstrating the superior quality of the point cloud in the proposed strategy compared to the SRP and SLP models. Table 2 lists the DMV and the measured error in different models. It reveals that the single-projector model fails to measure all the microgrooves, as illustrated in Figure 14 (**c**)–(**d**), where the point clouds in some microgroove areas are distorted and unsuitable for measurement. In contrast, the proposed strategy effectively measures all the microgrooves with a relatively stable range of error. However, for microgroove numbers 5–7, due to their reduced width and increased depth-width ratio (D/W), there are limitations imposed by the measurement principle of laser triangulation, which results in reduced measurement capabilities.

Based on the results of this experiment, we can conclude that the proposed strategy is capable of effectively measuring defects on high-reflectivity surfaces. It achieves accuracy of approximately 15 μm for small defects and exceeds 10 μm accuracy for relatively larger defects. The proposed strategy exhibits both higher measurement accuracy and robustness compared to the single-projector model, as evident from both MAE and PV.

### 3.3. Measuring High-Reflectivity Metal Plate

**Figure 16:**
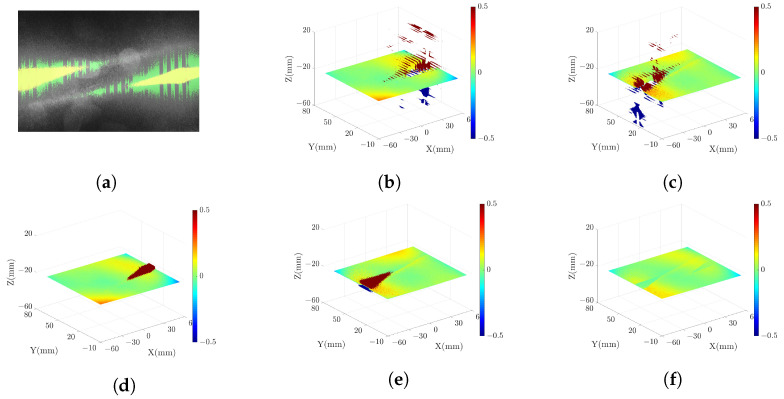
Scanning results of high-reflectivity metal plate: (**a**) grayscale image, (**b**) initial point cloud in SRP model, (**c**) initial point cloud in SLP model, (**d**) optimized point cloud in SRP model, (**e**) optimized point cloud in SLP model, (**f**) final fused point cloud. In (**b**–**f**), the color represents the distance from the point to the datum surface.

To verify the optimization of the point cloud for high-reflectivity surfaces, we conducted scans of a metal plate with the pattern parameters f={1,8,64} and N={4,4,96}. The scanning results are presented in Figure 16, with Figure 16 (**a**) displaying the grayscale image of the metal plate and Figure 16 (**b**)–(**f**) showing the point clouds under different conditions. In the grayscale image, the yellow and green pixels represent pixels in the initial point cloud identified as outliers. The outliers associated with green pixels are eliminated using the MPO method described in Section 2.3, while the outliers linked to yellow pixels are removed during the point cloud fusion process outlined in Section 2.4. Figure 16 (**f**) displays the point cloud without outliers.

To quantify the quality of the point cloud under different conditions, Table 3 lists the count of outliers deviating from the datum in different point clouds. The findings demonstrate a substantial reduction in outliers through the application of the MPO and point cloud fusion methods.

According to this experiment, we conclude that our strategy exhibits the capability of measuring a large range of high-reflectivity surfaces and generating accurate point clouds.

## 4. Conclusions

In this paper, we propose a novel quaternary categorization strategy for the scanning of high-reflectivity surfaces utilizing a setup consisting of two projectors and one camera. Firstly, we obtain two groups of phase information from two projectors along different directions, from which the pixels are classified into four types according to the phase characteristics. Secondly, we employ the MPO method to reduce the area of the invalid phase region. Based on the pixel type, we adaptively utilize either the DSLI model or the single-projector model for 3D reconstruction. Finally, we integrate the three categories of point clouds into one to obtain a high-accuracy result. The experimental results show that, in terms of point cloud accuracy, the DSLI model exhibits an enhancement of over 50% compared to the single-projector model; regarding micro-defect measurements, the quaternary categorization strategy achieves accuracy of approximately 15 μm, representing an improvement of over 40% compared to the single-projector model. Concerning the measurements of broad high-reflectivity surfaces, the quaternary categorization strategy effectively conducts measurements and mitigates outliers. In summary, our strategy significantly improves the measurement accuracy and robustness of SLI systems. In future work, we will further optimize our strategy to mitigate the impact of projector distortion on the accuracy of point cloud reconstruction. 

## Figures and Tables

**Table 1:** The errors of standard plane point clouds in different models.

Projection	DSLI Model (μm)	Single-Projector Model (μm)	
		SRP Model	SLP Model
MAE	4.55	8.83	9.7
PV	57.04	111.6	108.13
RMSE	5.65	11.53	12.38
Improvement ^a^	51.00% ^b^	54.36% ^c^	

^a^ The ratio of improvement in RMSE. ^b^ Compared with SRP model. ^c^ Compared with SLP model.

**Table 2:** The errors of microgroove point clouds in different models.

Microgroove Number	Ground Truth (μm)			Proposed Strategy (μm)		Single-Projector Model (μm)			
						SRP Model		SLP Model	
	Depth	Width	D/W	DMV	Error	DMV	Error	DMV	Error
1	297	1002	29.6%	290.8	−6.2	265.5	−31.5	UM ^a^	\
2	198	551	35.9%	189.1	−8.9	196.9	−1.1	UM ^a^	\
3	148	399	37.1%	146.1	−1.9	139.9	−8.1	UM ^a^	\
4	109	280	38.9%	97.5	−11.5	127.3	18.3	127	18.0
5	79	191	41.4%	63.9	−15.1	101.5	22.5	53.3	−25.7
6	59	130	45.9%	46.7	−12.3	81.2	22.2	72.2	13.2
7	49	100	49.0%	33.5	−15.5	UM ^a^	\	37.4	−11.6
PV	\			\	13.6	\	54.0	\	38.9
MAE	\			\	10.2	\	17.3	\	17.1
Improvement ^b^	40.70%								

^a^ The microgroove is unable to be measured. ^b^ The ratio of improvement in MAE.

**Table 3:** The number of outliers in different point clouds.

Figure Number	Figure 16 (b)	Figure 16 (c)	Figure 16 (d)	Figure 16 (d)	Figure 16 (f)
Outliers	1,772,479	1,414,155	622,677	852,861	0
Valid points	18,408,833	18,767,157	19,558,635	19,328,451	20,181,312
Valid ratio	91.22%	92.99%	96.91%	95.77%	100%

## Data Availability

The data are not publicly available due to personal privacy.
